# Vermittlung praktischer Fähigkeiten in der curricularen Lehre – Effekt von SkillsLab und „flipped classroom“

**DOI:** 10.1007/s00106-023-01408-5

**Published:** 2024-01-15

**Authors:** Judith Wehling, Tobias Dombrowski, Katharina Johannsen, Stefan Volkenstein, Stefan Dazert, Nora M. Weiss

**Affiliations:** 1 Klinik für Hals‑, Nasen- und Ohrenheilkunde, St. Elisabeth-Hospital, Ruhr-Universität Bochum, Bochum, Deutschland; 2 Klinik für Hals-Nasen-Ohrenheilkunde, Universitätsmedizin Göttingen, Göttingen, Deutschland; 3 Klinik für Hals‑, Nasen‑, Ohrenheilkunde der Ruhr-Universität Bochum, Johannes Wesling Klinikum Minden, Minden, Deutschland; 4 Klinik und Poliklinik für Hals‑, Nasen- und Ohrenheilkunde, Klinikum rechts der Isar der Technischen Universität München, München, Deutschland

**Keywords:** Hals-Nasen-Ohren-Heilkunde, Interaktives Lernen, Lehre, Medizinstudierende, Klinische Fähigkeiten, Otolaryngology, Interactive learning, Teaching, Medical students, Clinical skills

## Abstract

**Hintergrund:**

Im Zuge der Neustrukturierung des Medizinstudiums werden erstmalig auch praktische Kompetenzen als Lernziele klar definiert. Zur effektiven Nutzung der kurzen Präsenzzeit, die im Fach HNO-Heilkunde zur Verfügung steht, war das Ziel dieser Studie die Verzahnung der Vermittlung praktischer Fertigkeiten unter Zuhilfenahme von „flipped classroom“, digitaler Lehre und einem neu errichteten SkillsLab zu evaluieren.

**Material und Methoden:**

Im Rahmen des HNO-Praktikums wurden die Studierenden in 2 Gruppen unterteilt: Gruppe A = 93 Studierende (männlich *n* = 42, weiblich *n* = 51), Gruppe B = 113 Studierende (männlich *n* = 42, weiblich *n* = 71). Sie bearbeiteten zunächst digital zur Verfügung gestelltes Material, welches einzelne HNO-Untersuchungen erläuterte. Anschließend erfolgte der Präsenzunterricht in Kleingruppen, welcher eine Hospitation und Übungen der Untersuchungstechniken beinhaltete. Während Gruppe A diese dozentengeführt an Modellen absolvierte und sich gegenseitig untersuchte, erhielt Gruppe B den Unterricht anhand strukturierter Arbeitsstationen im hierfür neu errichteten HNO-SkillsLab. Die Effekte auf die Motivation und subjektiven Kompetenzen wurden anhand eines eigens für diese Studie entwickelten Fragebogens gemessen.

**Ergebnisse:**

Nach Bearbeitung des digitalen Materials zeigte sich in beiden Gruppen ein hohes Level an Motivation und Kompetenz. Im Verlauf des Präsenztags zeigte sich ein Zugewinn an Motivation und Kompetenz, welcher nur in der SkillsLab-Gruppe statistisch signifikant war (*p* < 0,001). Obwohl die SkillsLab-Gruppe vor dem Präsenztag in ihrer subjektiven Kompetenz unterlegen war, zeigte sie sich nach dem Präsenztag der anderen Gruppe hierin sogar überlegen.

**Schlussfolgerung:**

Die Kombination eines digitalen „flipped classroom“ mit strukturierten Arbeitsstationen im Rahmen eines SkillsLabs ermöglicht eine effektivere Vermittlung praktischer Kompetenzen, welche sich in der statistisch signifikanten Steigerung von Motivation und Selbsteinschätzung der Gruppe B widerspiegelt. Dabei erleichtert insbesondere die digitale Befunderhebung die Überprüfung der individuellen Lernerfolge und regt die Diskussion an.

**Zusatzmaterial online:**

Die Online-Version dieses Beitrags (10.1007/s00106-023-01408-5) enthält den Fragebogen zur Studie.

## Abstract (en)

**Background:**

In the course of the restructuring of medical studies, practical competencies are clearly defined as learning objectives for the first time. In order to make most effective use of the short attendance time available in otolaryngology, the aim of this study was to teach practical skills with the help of flipped classroom, digital teaching, and a newly established SkillsLab.

**Materials and methods:**

During their ENT internship, two groups of students—group A = 93 students (male *n* = 42, female *n* = 51) and group B = 113 students (male *n* = 42, female *n* = 71)—first worked through material provided online, which explained the individual examinations. This was followed by face-to-face teaching, which consisted of observation and practical exercise of the different examination techniques. While group A practiced on each other or on dummies, group B used structured workstations in the ENT SkillsLab, which was newly built for this purpose. The effects on motivation and subjective competence were measured using a questionnaire developed for this study.

**Results:**

After working through the online material, both groups showed a high level of motivation and competence. On the day of face-to-face teaching, there was a gain in motivation and competence, which was statistically significant only in the SkillsLab group (*p* < 0.001). Although the SkillsLab group was inferior in terms of its subjective competence at the beginning, it was superior to the other group after the face-to-face teaching.

**Conclusion:**

Combination of digitized teaching in the flipped classroom with structured workstations in the setting of a SkillsLab enables more effective teaching of practical skills, which was reflected by increases in motivation and subjective competence in group B. In particular, the presentation of all collected findings on monitors allows verification of learning success and stimulates discussion.

## Einleitung

Die Strukturierung des Medizinstudiums befindet sich in stetigem Wandel, sodass zuletzt durch die Einführung des Masterplans Medizinstudium 2020 [13] und des Nationalen Kompetenzbasierten Lernzielkatalogs Medizin (NKLM) 2.0 [10] der Fokus weiter auf das Erlernen praktischer Fähigkeiten ausgerichtet wurde, indem diese erstmalig als Lernziele mit Kompetenztiefe definiert wurden. Die Aneignung dieser Fertigkeiten ist zeitintensiv und kann i. d. R. nur ungenügend theoretisch bzw. außerhalb von Praktika erfolgen. Eine Neuausrichtung der curricularen Lehre mit besonderer Berücksichtigung manueller Fertigkeiten erscheint daher angesichts dieser Herausforderung notwendig.

In der Lehre der „kleinen“ Fächer, zu denen auch die Hals‑, Nasen‑, Ohren-Heilkunde gehört, ist weiterhin eine im Curriculum oft nur begrenzt vorgesehene Unterrichtszeit zu berücksichtigen. Im Gegensatz hierzu stehen jedoch die Lernziele des NKLM 2.0, welche für das Fach Hals‑, Nasen‑, Ohrenheilkunde eine studentische Handlungskompetenz in Bereichen fordert, die bisher der Weiterbildung vorbehalten waren, wie beispielsweise das Tamponieren einer Nase oder die Laryngoskopie [10].

SkillsLabs dienen dem Erlernen standardisiert aufgearbeiteter Fertigkeiten im Rahmen einer geschützten Lernumgebung. Dabei konnte bei der Vermittlung ärztlicher Fähigkeiten nachgewiesen werden, dass SkillsLabs sowohl zu einer objektiven (besseres Abschneiden der Interventionsgruppe in der OSCE-Prüfung, „objective structured clinical examination“) als auch zu einer subjektiven Verbesserung (subjektive Evaluation) der jeweiligen Handlungskompetenzen führen [11]. Im Rahmen der COVID-19-Pandemie erwiesen sich außerdem Versuche, praktische Fertigkeiten auch im Rahmen digital durchgeführter Lehrveranstaltungen zu vermitteln, als erfolgreich [8].

Eine weitere in der medizinischen Lehre etablierte Lehrmethode ist die Strategie des „flipped classroom“, in dessen Rahmen Lehrmaterial im Vorfeld zur Präsenzveranstaltung bearbeitet und gelernt wird, sodass die Präsenzzeit effektiv zur Rekapitulation, Analyse und praktischen Anwendung des Erlernten genutzt werden kann [3–5].

Unter Anwendung der genannten Lehrmethoden war das Ziel der geschilderten Arbeit die Neukonzeption des HNO-Praktikums an der Ruhr-Universität Bochum mit Fokussierung auf das Erlernen praktischer Fähigkeiten. Dabei sollten die Anforderungen des Masterplans Medizinstudium 2020 und des NKLM 2.0 berücksichtigt sowie die zur Verfügung stehende, knappe Praktikumszeit durch im Rahmen von „flipped classroom“ präsentierte, digitale Inhalte und ein neu eingerichtetes HNO-SkillsLab möglichst effektiv genutzt werden. Der Effekt der Präsenzlehre sowie des SkillsLabs sollte anhand der Motivation und der subjektiven Kompetenzen der Studierenden gemessen werden.

## Material und Methoden

### Stichprobe

**Figure 1:**
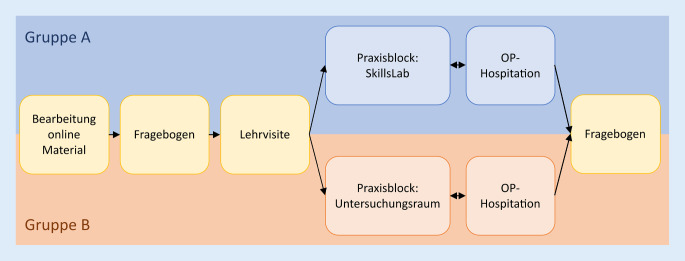

Diese Studie wurde mit einem Kollektiv von 206 Studierenden der Humanmedizin des 9. Semesters durchgeführt, welche am Blockpraktikum Hals-Nasen-Ohren-Heilkunde der Ruhr-Universität Bochum im Wintersemester 2021/2022 und Sommersemester 2022 teilnahmen. Das Kollektiv wurde in eine Kontrollgruppe (Gruppe A) von 93 Studierenden (männlich *n* = 42, weiblich *n* = 51), die zu Beginn des Semesters in das HNO-Praktikum eingeteilt waren, und eine Interventionsgruppe (Gruppe B) von 113 Studierenden (männlich *n* = 42, weiblich *n* = 71), die in der zweiten Hälfte des Semesters für das Praktikum eingeteilt waren, unterteilt (Abb. 1). Daten bezüglich Studierender, die sich möglicherweise im Zweitstudium befinden, lagen nicht vor.

### Praktikumsplanung

Das curriculare HNO-Praktikum der Ruhr-Universität Bochum besteht aus 2 Tagen mit jeweils 5 Unterrichtsstunden, welche in Kleingruppen von maximal 6 Studierenden absolviert werden. Die Inhalte des Praktikums wurden zunächst inhaltlich auf die praktischen Fertigkeiten, welche im NKLM 2.0 für das Fach Hals-Nasen-Ohren-Heilkunde gefordert werden, abgestimmt. Um die knappe Zeit der Präsenzlehre dabei so effektiv wie möglich zu gestalten, wurde das Praktikum weitergehend in 2 methodisch voneinander getrennte Abschnitte unterteilt: Tag 1 und Tag 2.

#### Tag 1: Online-Material als „digital flipped classroom“

**Figure 2:**
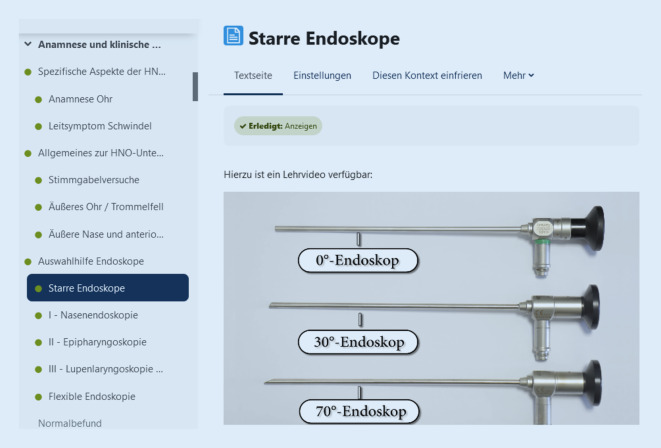

Aufgrund der COVID-19-Pandemie wurde seitens des Studiendekanats ein möglichst verdichteter Präsenzunterricht gewünscht, sodass Tag 1 des HNO-Praktikums als reine Online-Veranstaltung geplant wurde. Da in vorherigen Praktika ein geringes praktisches Vorwissen der Studierenden aufgefallen war, sollte das online dargebotene Material nach dem Prinzip von „flipped classroom“ Grundkenntnisse verschaffen und auf die praktischen Übungen an Tag 2 vorbereiten. Um den Studierenden hierbei Freiraum in Bezug auf Geschwindigkeit und Zeitpunkt der Bearbeitung zu gewähren, wurde eine asynchrone Veranstaltung als Mittel gewählt. In diesem Kontext wurden alle Materialien hinsichtlich ihrer praktischen Relevanz selektiert und anschließend auf der Plattform Moodle in Form von Lehrvideos, Texten, interaktiven Grafiken und klinischen Fällen zur Verfügung gestellt (Abb. 2). Inhaltlich bildeten diese die Themen Anamnese, allgemeine Anatomie, Ohrmikroskopie, anteriore Rhinoskopie, flexible und starre Endoskopie, Laryngoskopie und das Interpretieren eines Reintonaudiogramms ab. Darüber hinaus fungierten klinische Fallbeispiele als Lernkontrolle. Abschließend mussten alle Studierenden einen Fragebogen bearbeiten, um zum Tag 2 in Form von Präsenzunterricht zugelassen zu werden.

#### Fragebogen

Im Rahmen der Befragung sollte die Selbsteinschätzung der Studierenden im Laufe des HNO-Praktikums abgebildet werden. Hierfür wurde von den Studierenden ein eigens hierfür entwickelter, nicht validierter Fragebogen (Zusatzmaterial online) ausgefüllt, welcher neben personenbezogenen Daten im Rahmen von 38 Items die Themenbereiche Motivation (z. B. „Ich finde das Fach HNO interessant.“) und die Selbsteinschätzung hinsichtlich HNO-spezifischer Kompetenzen (z. B. „Ich fühle mich in der Lage, eine HNO-Untersuchung durchzuführen.“, „Ich traue mir zu, einen Paukenerguss zu erkennen.“) erfasst. Alle Items wurden in Form einer 5‑stufigen Likert-Skala abgefragt („trifft sehr zu“, „trifft zu“, „trifft nicht zu“, „trifft überhaupt nicht zu“, „weiß nicht“). Dabei wurden für die jeweilige Antwort zwischen 0 („trifft überhaupt nicht zu“) und 3 („trifft sehr zu“) Punkten vergeben. Außerdem wurden eine Gesamtnote (Schulnoten 1–6) für das Praktikum sowie die Freitextrubriken „Das würde ich besser machen.“ und „Das hat mir gut gefallen.“ abgefragt. Der identische Fragebogen wurde an Gruppe A und Gruppe B jeweils nach Tag 1 sowie nach Tag 2 angewendet.

#### Tag 2: Präsenzunterricht, Üben praktischer Fähigkeiten

In beiden Armen der Studie wurden die Gruppen zu Beginn des Präsenztags in zwei 3er-Gruppen unterteilt und anschließend auf 2 gemischte HNO-Stationen verteilt, auf denen sich jeweils Patienten mit Tumor‑, Infektions- und allgemeinen HNO-Erkrankungen befinden. In diesem Rahmen fand für jede Gruppe eine Lehrvisite von etwa einer Stunde Dauer statt, in deren Rahmen Krankheitsbilder, klinische Befunde und Untersuchungstechniken diskutiert und demonstriert wurden. Anschließend fand für eine der 3er-Gruppen eine etwa 90-minütige Hospitation im Operationssaal (OP) statt, während die andere 3er-Gruppe einen etwa zweieinhalbstündigen Praxisblock durchlief. Anschließend wurden die Gruppen getauscht, sodass alle Studierenden dieselben Stationen durchliefen.

**Figure 3:**
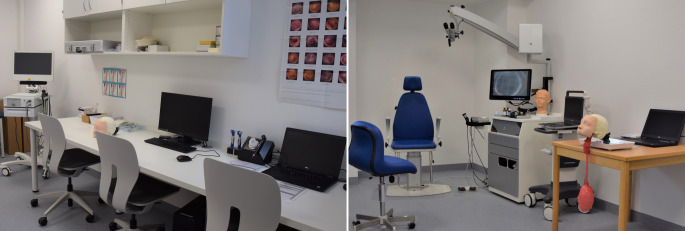

Die Intervention der geschilderten Studie bestand in der unterschiedlichen Gestaltung des Praxisblocks während des Präsenztags (Tab. 1). Inhaltlich sollten dabei in allen Gruppen folgende Themen abgedeckt und durch die an der Lehre beteiligten AssistenzärztInnen strukturiert vermittelt werden: Mundinspektion, anteriore Rhinoskopie, Ohrmikroskopie, starre und flexible Endoskopie, Reintonaudiometrie und otoskopische Befunde, Legen einer Magensonde, Nasentamponade und Koniotomietechniken. Um hierbei ein möglichst standardisiertes Vorgehen zu gewährleisten, wurden alle an der Lehre beteiligten AssistenzärztInnen vor Beginn des Praktikums in die einzelnen Übungsstationen eingewiesen. Ferner wurden jedem Dozierenden detailreiche Ablaufpläne für jede Übungsstation ausgehändigt. Während Gruppe A diese Inhalte im Behandlungszimmer entweder durch gegenseitige Untersuchung oder an verschiedenen Modellen beigebracht wurden, wurde Gruppe B im eigens hierfür eingerichteten SkillsLab (Abb. 3) an Arbeitsstationen mit fest vorgegebenem Ablauf unterrichtet. Sowohl der Kontroll- als auch der Interventionsgruppe standen dabei verschiedene Modelle zu Übungszwecken zur Verfügung. Während die Studierenden der Gruppe A an diesen Modellen üben konnten, wurde in Gruppe B ein Teil der Untersuchung zusätzlich auf Bildschirme übertragen und konnte so in der Gruppe erläutert und diskutiert werden. Außerdem wurden die Übungen im SkillsLab um ein Koniotomie- und ein Ohrmodell erweitert.

**Table 1:** 

Inhalte Praxisblock	Gruppe A	Gruppe B
Mundinspektion	Gegenseitig	Gegenseitig
Anteriore Rhinoskopie	Gegenseitig/Modell	Gegenseitig/Modell
Ohrmikroskopie/Otoskopie	Gegenseitig	Gegenseitig/Modell
Legen einer Magensonde	Modell	Modell
Starre und flexible Endoskopie	Gegenseitig/Modell	Gegenseitig/Modell mit Darstellung auf Monitor
Tamponade einer Nase	Modell	Modell
Audiometriebeispiele und otoskopische Befunde	Audiometriebeispiele mit Ohrbefunden als Bild	Audiometriebeispiele mit Ohrbefunden am Otoskopiemodell
Koniotomie	Demonstration verschiedener Koniotomiesets	Benutzung verschiedener Koniotomiesets am Modell

Nach dem Präsenztag mussten alle Studierenden den genannten Fragebogen erneut bearbeiten, um das Praktikum abschließen zu können.

#### Statistische Auswertung

Die Statistische Auswertung der Daten erfolgte mit dem Statistikprogramm SPSS 28.0.1.1. (Fa. IBM Corp., Armonk/NY, USA, IBM SPSS Statistics for Macintosh, Version 28.0.1.1. ) und Microsoft® Excel® 2016 MSO (Version 2306 Build 16.0.16529.20164, 64 Bit, Redmond/WA, USA). Der Vergleich der Zeitpunkte und Gruppen wurde mithilfe einer 2‑faktoriellen Varianzanalyse (ANOVA) sowie eines korrigierten t‑Tests als Post-hoc-Analyse angewendet. Das Signifikanzniveau wurde mit *p* < 0,05 festgelegt.

### Ergebnisse

Während am Online-Praktikum (Tag 1) 92 (Gruppe A) bzw. 113 (Gruppe B) Studierende teilnahmen, absolvierten 80 (Gruppe A) bzw. 101 (Gruppe B) Studierende das Präsenzpraktikum (Tag 2). Studierende, die nicht am Präsenztag teilnahmen, fielen i. d. R. durch eine Krankschreibung aus.

Das gemittelte Alter der Studierenden während des Online-Praktikums an Tag 1 betrug in Gruppe A (= A1) 25,23 Jahre bzw. in Gruppe B (= B1) 26,75 Jahre. Bei der Durchführung des Präsenztags an Tag 2 betrug das gemittelte Alter 24,58 Jahre in Gruppe A (= A2) und 26,71 Jahre in Gruppe B (= B2).

Dabei bewerteten die Studierenden das Praktikum an Tag 1 mit einer Gesamtnote von 1,75 (A1) bzw. 1,95 (B1) sowie an Tag 2 mit einer Gesamtnote von 1,65 (A2) bzw. 1,6 (B2). In der Rubrik „Das würde ich besser machen.“ wurde in beiden Gruppen mehr Präsenzunterricht zusätzlich zur Bearbeitung des Online-Materials gewünscht, während in der Rubrik „Das hat mir gut gefallen.“ in beiden Gruppen eine angenehme Atmosphäre sowie in Gruppe B das SkillsLab positiv bewertet wurden.

#### Motivation

**Figure 4:**
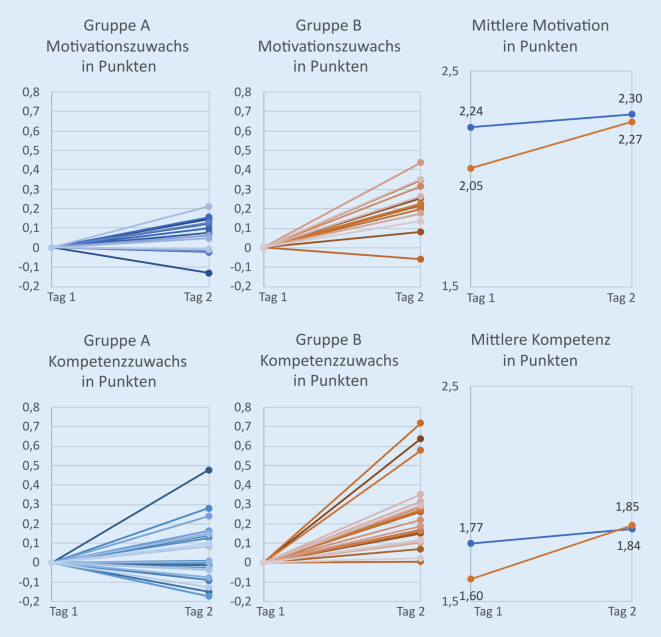

Nach Tag 1 zeigte sich die mittlere Motivation in der Interventionsgruppe (Gruppe B) statistisch signifikant (*p* < 0,001) geringer als die der Kontrollgruppe (Gruppe A), obwohl beide denselben Online-Kurs bearbeitet hatten. Anschließend stieg die Motivation zwischen Tag 1 und Tag 2 in beiden Gruppen an. Dabei zeigte sich der Zugewinn an Motivation in Gruppe A von 2,24 Punkten an Tag 1 (Standardabweichung 0,35) auf 2,3 Punkte (Std.-Abweichung 0,39) an Tag 2 mit 0,06 Punkten nicht statistisch signifikant (*p* = 0,315). In der Interventionsgruppe (Gruppe B) zeigte sich mit einem mittleren Motivationsanstieg von 0,22 Punkten eine statistische Signifikanz (*p* < 0,001) mit einem Anstieg der Motivation von 2,05 Punkten (Std.-Abweichung 0,42) an Tag 1 auf 2,27 Punkte (Std.-Abweichung 0,32) an Tag 2 (Abb. 4). Obwohl Gruppe B an Tag 1 in jedem Motivations-Item weniger Punkte angab als Gruppe A, glich sie sich nach Tag 2 in ihrer Motivation der Gruppe A an und bewertete sogar 3 Motivations-Items, welche sich alle mit dem generellen Nutzen eines SkillsLabs beschäftigten, besser als Gruppe A („Ich finde Lehre in SkillsLabs sinnvoll.“, „Von im SkillsLab vermittelten Kompetenzen werde ich in meinem Berufsleben profitieren können.“, „Ein SkillsLab ist zur Vertiefung der zuvor erlernten, theoretischen Inhalte sinnvoll.“). Statistisch betrachtet bestand so nach Tag 2 insgesamt kein signifikanter Motivationsunterschied mehr zwischen Gruppe A und B.

#### Kompetenzen

Auch in der Selbsteinschätzung der eigenen Kompetenzen unterschieden sich beide Gruppen an Tag 1 signifikant voneinander (*p* = 0,006). Dabei schätzte sich Gruppe A mit im Mittel 1,77 Punkten (Std.-Abweichung 0,45) insgesamt besser ein als Gruppe B mit im Mittel 1,6 Punkten (Std.-Abweichung 0,41). Nach dem Präsenztag schätzten sich beide Gruppen kompetenter ein, wobei Gruppe B das Kompetenzlevel von Gruppe A übertraf (A2 = 1,84; B2 = 1,86), sodass an Tag 2 kein statistischer Unterschied mehr zwischen den subjektiven Kompetenzen der beiden Gruppen zu erkennen war (*p* = 0,79; Abb. 4).

Dabei lag der Kompetenzzuwachs zwischen Tag 1 und Tag 2 im Mittel bei 0,07 Punkten in Gruppe A und 0,25 Punkten in Gruppe B. Nur in Letzterer erwies sich der Kompetenzzugewinn als statistisch signifikant (*p* < 0,001). Während sich Gruppe B nach Tag 1 in nur 2 von 25 Kompetenz-Items besser eingeschätzt hatte als Gruppe A, lag sie nach Abschluss des Praktikums (Tag 2) bei 17 Items höher als Gruppe A. In Gruppe B kam es weiterhin bei keinem Kompetenz-Item zu einer Verschlechterung zwischen Tag 1 und Tag 2, wohingegen die subjektive Kompetenz in Gruppe A in 12 Items abnahm. Die größte Differenz an Kompetenzzugewinn in Gruppe B erzielten die Items „Ich traue mir zu, eine Nasenendoskopie durchzuführen und die wichtigsten anatomischen Landmarken zu benennen.“ (0,45 Punkte), „Ich traue mir zu, eine anteriore Rhinoskopie durchzuführen und die wichtigsten anatomischen Landmarken zu benennen.“ (0,44 Punkte), „Ich traue mir zu, eine Stimmlippenparese zu erkennen.“ (0,39 Punkte), „Ich traue mir zu, einen Hals zu untersuchen und die wichtigsten anatomischen Landmarken zu benennen.“ (0,33 Punkte) und „Ich traue mir zu, einen Paukenerguss zu erkennen.“ (0,3 Punkte).

## Diskussion

Der NKLM 2.0 rückt den Fokus des Medizinstudiums stärker und frühzeitig auf praktisch durchzuführende Maßnahmen und definiert zu diesem Zweck die Kompetenzebenen 1 bis 3. Die Kompetenzebene 3 steht dabei für Handlungskompetenz und wird in 3a „unter Anleitung selbst durchführen und demonstrieren“ und 3b „selbstständig und situationsadäquat in Kenntnis der Konsequenzen durchführen“ unterteilt [10]. Als für das Fach Hals‑, Nasen‑, Ohrenheilkunde spezifische Anforderungen gibt der NKLM ein Kompetenzlevel 3a in den Fähigkeiten „eine Laryngoskopie durchführen“, „Spontan- und Provokationsnystagmus mit der Frenzel-Brille prüfen“ und „eine Nase tamponieren“ an. Weitergehend sollen die Kompetenzen „klinisch-apparative Untersuchung des Ohrs“, „anteriore Rhinoskopie“ und die „Stimmgabelprüfung nach Weber und Rinne“ nach Kompetenzlevel 3b beherrscht werden.

Zur Vermittlung verschiedener praktischer Fertigkeiten sind Ansätze wie Simulationen an Modellen oder Patienten in SkillsLabs und verschiedene digitale Lehrangebote verbreitet. Dabei wurde sowohl die Effektivität digitaler Konzepte als auch die von Simulationen in unterschiedlichen Formen nachgewiesen [8, 9]. So führte beispielsweise die digitale Vermittlung der HNO-Untersuchung zu einem signifikanten Leistungszuwachs bei Studierenden der Humanmedizin [8] sowie die Übung praktischer Fertigkeiten an virtuellen und physischen Modellen zu einer größeren klinischen Kompetenz, einer gesteigerten Geschicklichkeit und Sicherheit beim Umgang mit dem Instrumentarium bei AssistenzärztInnen [6, 9]. Unklar ist jedoch, wie eine effektive Kombination beider Systeme sowie deren Implementierung in die curriculare HNO-Lehre gestaltet werden kann.

Im Rahmen der geschilderten Studie wurde daher das HNO-Praktikum der Ruhr-Universität Bochum unter besonderer Berücksichtigung der praktisch zu erlernenden Fähigkeiten umstrukturiert und neu ausgerichtet. Dabei sollte die sehr begrenzte zur Verfügung stehende Zeit von 2 Tagen, durch die Kombination eines „digitalen flipped classroom“ und eines HNO-SkillsLabs möglichst effektiv gestaltet werden.

Auch wenn das Erlernen praktischer Fähigkeiten letztendlich manuell geübt werden muss, zeigt die Anwendung von E‑Learning im Sinne von „flipped classroom“ in dieser Untersuchung großes Potenzial. Die Bereitstellung von Online-Material zur Bearbeitung vor dem Präsenztag bietet dabei den Vorteil, von den Studierenden überall, zu jedem Zeitpunkt und im eigenen Tempo bearbeitet werden zu können. Zusätzlich garantiert ein verpflichtender, standardisierter Online-Kurs vor der Durchführung des Praktikums einen ähnlichen Wissensstand aller Studierenden zum Zeitpunkt des Präsenzunterrichts und vereinfacht so die Unterrichtsplanung für Dozierende deutlich.

### Tag 1 online

In ihrer Übersichtsarbeit beschreiben Bugaj et al. die Vorbereitung auf das Training praktischer Fertigkeiten mittels theoretischer Inhalte als einen wesentlichen Faktor, um den Lerneffekt zu steigern und die meist begrenzte Praktikumszeit möglichst effektiv zu nutzen [2]. Im Rahmen des ersten Praktikumstags wurden die Studierenden daher mit den online zur Verfügung gestellten Daten im Rahmen des „Flipped-Classroom-Konzepts“ in die praxisrelevanten Aspekte der HNO-Untersuchung eingeführt, Untersuchungstechniken wurden erläutert und demonstriert, womit der Wissensstand der Studierenden vor den praktischen Übungen angehoben und auf ein vergleichbares Level gebracht werden sollte. Anschließend zeigte sich das Motivationslevel mit einer Punktzahl von > 2 in beiden Gruppen relativ hoch (Maximalwert 3).

Unter Berücksichtigung der Tatsache, dass an Tag 1 alle praktischen Kompetenzen ausschließlich theoretisch erläutert und demonstriert wurden und ein Punktwert von 2 (bzw. 3) bedeutet, dass sich alle Studierenden alle Kompetenzen zutrauen (bzw. sehr zutrauen), zeigte sich auch die subjektive Kompetenz mit 1,77 Pkt. in Gruppe A und 1,6 Pkt. in Gruppe B erfreulich hoch. Dieses Ergebnis ist mit aktuellen Studiendaten vereinbar, da beispielsweise Kraus et al. einen signifikanten Leistungszuwachs Studierender durch einen online vermittelten HNO-Untersuchungskurs nachwiesen [8].

Dennoch fiel auf, dass sich Gruppe A und B nach Tag 1 sowohl in ihrer Motivation als auch in ihrer subjektiven Kompetenz voneinander unterschieden. So zeigte sich, dass Gruppe B in beiden Qualitäten unterlegen war, obwohl exakt dasselbe Online-Praktikum bearbeitet wurde. Es ist also anzunehmen, dass sich die Gruppen bereits vor dem Praktikum hinsichtlich Motivation und Kompetenz unterschieden, sodass bereits vor Beginn des Praktikums niedrigere Ausgangswerte in Gruppe B vorgelegen haben könnten. Hierfür spricht, dass die Studierenden nicht randomisiert wurden, sondern nach der zeitlichen Mitte des Semesters in Gruppe A und B unterteilt wurden. Dies führte dazu, dass Gruppe B mehr Studierende beinhaltete, welche Teile des Semesters nachholten, Urlaubssemester eingelegt hatten oder aktuell ihr Erasmus-Studium an der Ruhr-Universität Bochum absolvierten.

Alternativ hierzu wäre ein Beginn beider Gruppen auf gleichem Niveau vor Durchführung des Praktikums mit einem anschließenden, ungleichen Zugewinn an Motivation und Kompetenz während des Online-Praktikums durch Unterschiede innerhalb der Gruppen zu erwägen. Eine mögliche Ursache hierfür könnte das unterschiedliche Durchschnittsalter der beiden Gruppen sein, woraus geschlussfolgert werden könnte, dass jüngere Studierende von dem Online-Angebot mehr profitierten als ältere.

Unabhängig von der Ursache der unterschiedlichen Werte nach Tag 1 lässt sich jedoch zusammenfassen, dass das Online-Praktikum als eine gute Vorbereitung gewertet werden kann, welche zu erfreulich hohen Werten in Motivation und subjektiver Kompetenz führte sowie den zusätzlichen Nutzen eines ähnlichen Kenntnisstands aller Studierenden zu Beginn des Präsenztags zeigte. Dennoch konnte der evtl. vorbestehende Unterschied an Selbsteinschätzung durch das Online-Material nicht angeglichen werden. Die Integration online angebotener Lehrmaterialien ermöglicht dabei grundsätzlich eine ökonomischere Curriculumplanung hinsichtlich des von Dozentenseite zu investierenden Zeitaufwands [8]. Als Limitation ist zu erwähnen, dass eine zuverlässige Prüfung der Quantität der Nutzung des Vorbereitungsmaterials nicht sicher möglich war. Es wurde zwar überprüft, dass alle Studierenden die kompletten, online zur Verfügung gestellten Inhalte bearbeiteten, es wurde jedoch nicht kontrolliert, wie intensiv bzw. aufmerksam dies erfolgte.

### Tag 2 Präsenzunterricht

Die Befragung nach Tag 2 des Praktikums zeigte in beiden Gruppen einen Zuwachs an Motivation und Kompetenz. Statistisch signifikant war dieser jedoch nur in Gruppe B, welche eine standardisierte Ausbildung im neu eingerichteten SkillsLab erhielt. Hier zeigte sich ein mittlerer Zuwachs von 0,22 Punkten in der Motivation und 0,25 Punkten in der subjektiven Kompetenz.

Zu beachten ist hierbei, dass die vermittelten Kompetenzen in beiden Gruppen inhaltlich identisch waren (Tab. 1) und sich lediglich in der Form der Vermittlung unterschieden. Insbesondere wurden die im SkillsLab zu vermittelnden Untersuchungstechniken auf einzelne Übungsstationen aufgeteilt sowie deren Ablauf mittels Checklisten und vorheriger Einweisung der Dozierenden standardisiert. Durch die Anwendung solcher standardisierten Abläufe kann dabei trotz wechselnder Tutoren die Vollständigkeit der geübten Untersuchung garantiert werden sowie Lehrinhalte und Lernziele dozentenunabhängig definiert werden [7]. Auch unterschiedliche didaktische Geschicke verschiedener Dozenten können durch solche Standards ausgeglichen werden [7]. Weiterer Wirksamkeitsfaktor zur Lehre in simulierten Umgebungen ist die unmittelbare Rückmeldung durch Dozenten unmittelbar während des Trainings [1], sodass möglichst alle zu erhebenden Befunde auf Monitore übertragen wurden. Hierdurch zeigte sich aufseiten der Dozierenden die Vermittlung der Inhalte erleichtert, da z. B. live nachverfolgt werden konnte, ob die Studierenden tatsächlich die richtige anatomische Struktur identifiziert hatten. War dies nicht der Fall, konnte gemeinsam korrigiert oder im Team erörtert werden, um welche Struktur es sich alternativ handeln könnte. Mögliche Missverständnisse wurden hierdurch reduziert. Darüber hinaus konnte hierdurch innerhalb der Gruppe besser diskutiert und demonstriert werden.

Das SkillsLab als gesonderter Raum sorgte dabei für eine kontrollierte Umgebung, in der Untersuchungstechniken geübt werden konnten, ohne Patienten zu gefährden, was in aktueller Literatur ebenfalls als Erfolgsfaktor der praktischen Ausbildung gewertet wird [2, 9].

Bei der Betrachtung der einzelnen Kompetenz-Items zeigte sich, dass es in Gruppe A bei manchen Items zu einem subjektiven Kompetenzzuwachs kam, während sich die Studierenden der Gruppe A bei anderen Kompetenzen nach dem Präsenztag sogar schlechter einschätzten als zuvor. Im Gegensatz hierzu zeigte sich dieser Effekt in Gruppe B nicht, sodass sich die Studierenden der Gruppe B im Mittel an Tag 2 in jedem Item besser einschätzten als an Tag 1. Dies betraf interessanterweise auch Kompetenzen, die im Rahmen des Praktikums nicht direkt vermittelt wurden, wie beispielsweise „Ich traue mir zu, die HINTS auszuführen und auszuwerten.“ (HINTS-Methode: Head-Impulse-Test, d. h. Kopfimpulstest; Nystagmus; Test of Skew, d. h. „skew deviation“) oder „Ich traue mir zu, einen BPLS zu diagnostizieren.“ (benigner paroxysmaler Lagerungsschwindel).

Dies führte dazu, dass obwohl sich Gruppe B an Tag 1 sowohl hinsichtlich Motivation als auch subjektiver Kompetenz signifikant schlechter einschätzte als Gruppe A, es nach Tag 2 zu einer Angleichung beider Gruppen kam, wobei Gruppe B Gruppe A im Rahmen der subjektiven Kompetenz sogar geringfügig überholte und es nach dem Präsenztag keinen statistisch signifikanten Unterschied mehr zwischen den Kompetenzen der beiden Gruppen gab. Insgesamt lässt sich also festhalten, dass beide Gruppen vom Präsenztag und der Lehre im SkillsLab profitierten, die initial weniger motivierte und weniger kompetente Gruppe jedoch insgesamt stärkere Fortschritte erzielte als die andere. Trotz des hiermit verbundenen größeren persönlichen Zeitaufwands wünschten sich beide Gruppen mehr Präsenzlehre zusätzlich zum Online-Angebot.

## Limitationen

Die Limitationen der geschilderten Studie werden durch den jeweiligen Zeitpunkt der Befragung definiert. Um herauszufinden, ob die beiden Gruppen sich schon vor Beginn des Praktikums unterschieden, hätte eine weitere Befragung unmittelbar vor Beginn des Online-Praktikums durchgeführt werden müssen. Auch eine erneute Befragung mit zeitlichem Abstand nach dem Praktikum wäre in Bezug auf den bleibenden Lerneffekt interessant. Außerdem lässt das Studiendesign keine Differenzierung zwischen dem Effekt von Präsenzlehre und dem Effekt des SkillsLabs in Gruppe B zu. Auch hierfür hätte noch eine weitere Befragung stattfinden müssen. Da die Studierenden jedoch bereits im Rahmen der jetzigen Studie das hohe Maß an Evaluationen anmerkten, wurde hierauf verzichtet. Da die Gruppen nicht randomisiert, sondern nach zeitlicher Mitte des Praktikums aufgeteilt wurden, besteht zusätzlich ein Selektionsbias in Hinblick auf Alter und Geschlecht der Teilnehmer. Ferner handelt es sich bei der Bewertung der Motivation und der Kompetenzen ausschließlich um subjektive Selbsteinschätzungen der Studierenden. Wie jedoch Störmann et al. in ihrer Studie nachwiesen, schätzten nur ein Drittel aller Studierenden ihre eigenen praktischen Fähigkeiten richtig ein, während sich etwa 50 % um etwa 10–20 % überschätzten [12]. Um hier objektive Daten zu erheben und eine Selbstüberschätzung zu vermeiden, hätte beispielsweise eine „objective structured clinical examination“ (OSCE) eingeführt werden müssen, welche jedoch aufgrund der sehr begrenzten Praktikumszeit nicht realisiert werden konnte.

## Fazit für die Praxis

- Die Vermittlung praktischer Fertigkeiten ist bis zu einem gewissen Grad auch digital möglich, ersetzt aber nicht die Präsenzlehre.
- Die Effektivität der Präsenzlehre und insbesondere das Erlernen praktischer Fertigkeiten wird durch die Anwendung standardisierter Übungsstationen in SkillsLabs gesteigert.
- Mögliche Unterschiede in praktischen Kompetenzen lassen sich durch Präsenzlehre und SkillsLabs angleichen.

### Supplementary Information




