# Supplementary material for: Providing practical skills in curricular teaching—effect of SkillsLab and flipped classroom
Source: HNO. 2024 Jan 15;72(3):143–53. [Article in German] doi: 10.1007/s00106-023-01408-5 (PMC10879220; doi:10.1007/s00106-023-01408-5)
Supplement: Supplementary file 1 [file 106_2023_1408_MOESM1_ESM.pdf]

Fragebogen: HNO-Praktikum 2021/2022

Alter:____________________

Geschlecht:______________________

Ich finde das Fach HNO interessant

☐Trifft sehr zu ☐Trifft zu ☐Trifft nicht zu ☐Trifft überhaupt nicht zu ☐Weiß nicht

Ich finde eLearning sinnvoll

☐Trifft sehr zu ☐Trifft zu ☐Trifft nicht zu ☐Trifft überhaupt nicht zu ☐Weiß nicht

Ich finde Lehre in Skills Labs sinnvoll

☐Trifft sehr zu ☐Trifft zu ☐Trifft nicht zu ☐Trifft überhaupt nicht zu ☐Weiß nicht

Das HNO-Praktikum ist sinnvoll aufgebaut.

☐Trifft sehr zu ☐Trifft zu ☐Trifft nicht zu ☐Trifft überhaupt nicht zu ☐Weiß nicht

Das online-Praktikum ist sinnvoll aufgebaut.

☐Trifft sehr zu ☐Trifft zu ☐Trifft nicht zu ☐Trifft überhaupt nicht zu ☐Weiß nicht

Ich habe in dieser Veranstaltung viel gelernt

☐Trifft sehr zu ☐Trifft zu ☐Trifft nicht zu ☐Trifft überhaupt nicht zu ☐Weiß nicht

Ich kann wesentliche Inhalte der Veranstaltung erläutern

☐Trifft sehr zu ☐Trifft zu ☐Trifft nicht zu ☐Trifft überhaupt nicht zu ☐Weiß nicht

Ich fühle mich in der Lage das im online-Praktikum Erlernte auch praktisch anzuwenden

☐Trifft sehr zu ☐Trifft zu ☐Trifft nicht zu ☐Trifft überhaupt nicht zu ☐Weiß nicht

Von den im Skillslab vermittelten Kompetenzen werde ich in meinem Berufsleben profitieren können.

☐Trifft sehr zu ☐Trifft zu ☐Trifft nicht zu ☐Trifft überhaupt nicht zu ☐Weiß nicht

Präsenzunterricht ist zur Vertiefung der erlernten Inhalte sinnvoll.

☐Trifft sehr zu ☐Trifft zu ☐Trifft nicht zu ☐Trifft überhaupt nicht zu ☐Weiß nicht

Ein Skills Lab ist zur Vertiefung der zuvor erlernten, theoretischen Inhalte sinnvoll

☐Trifft sehr zu ☐Trifft zu ☐Trifft nicht zu ☐Trifft überhaupt nicht zu ☐Weiß nicht

Gemessen am meinem Lernerfolg finde ich den Arbeitsaufwand angemessen

☐Trifft sehr zu ☐Trifft zu ☐Trifft nicht zu ☐Trifft überhaupt nicht zu ☐Weiß nicht

Das online-Praktikum hat mich neugierig auf eine weiterführende Auseinandersetzung mit einzelnen Lehrinhalten gemacht

☐Trifft sehr zu ☐Trifft zu ☐Trifft nicht zu ☐Trifft überhaupt nicht zu ☐Weiß nicht

Gesamtnote für das HNO-Praktikum (1 - 6): ___

Das würde ich besser machen:

Das fand ich gut:

Selbsteinschätzung

Ich fühle mich in der Lage eine HNO-Untersuchung durchzuführen.

☐Trifft sehr zu ☐Trifft zu ☐Trifft nicht zu ☐Trifft überhaupt nicht zu ☐Weiß nicht

Ich traue mir zu die wichtigsten anatomischen Landmarken des Ohres zu benennen.

☐Trifft sehr zu ☐Trifft zu ☐Trifft nicht zu ☐Trifft überhaupt nicht zu ☐Weiß nicht

Ich traue mir zu ein Trommelfell mithilfe eines Ohr-Mikroskop zu untersuchen.

☐Trifft sehr zu ☐Trifft zu ☐Trifft nicht zu ☐Trifft überhaupt nicht zu ☐Weiß nicht

Ich traue mir zu eine Otits media acuta/ Otits externa zu erkennen.

☐Trifft sehr zu ☐Trifft zu ☐Trifft nicht zu ☐Trifft überhaupt nicht zu ☐Weiß nicht

Ich traue mir zu einen Paukenerguss zu erkennen.

☐Trifft sehr zu ☐Trifft zu ☐Trifft nicht zu ☐Trifft überhaupt nicht zu ☐Weiß nicht

Ich traue mir zu einen orientierenden Hörtest mit Weber und Rinne durchzuführen und zu bewerten.

☐Trifft sehr zu ☐Trifft zu ☐Trifft nicht zu ☐Trifft überhaupt nicht zu ☐Weiß nicht

Ich traue mir zu einen Spontannystagmus zu erkennen.

☐Trifft sehr zu ☐Trifft zu ☐Trifft nicht zu ☐Trifft überhaupt nicht zu ☐Weiß nicht

Ich traue mir zu die HINTS auszuführen und auszuwerten.

☐Trifft sehr zu ☐Trifft zu ☐Trifft nicht zu ☐Trifft überhaupt nicht zu ☐Weiß nicht

Ich traue mir zu einen BPLS zu diagnostizieren.

☐Trifft sehr zu ☐Trifft zu ☐Trifft nicht zu ☐Trifft überhaupt nicht zu ☐Weiß nicht

Ich traue mir zu die wichtigsten anatomischen Landmarken der Nase zu benennen.

☐Trifft sehr zu ☐Trifft zu ☐Trifft nicht zu ☐Trifft überhaupt nicht zu ☐Weiß nicht

Ich traue mir zu eine anteriore Rhinoskopie durchzuführen und die wichtigsten anatomischen Landmarken zu benennen.

☐Trifft sehr zu ☐Trifft zu ☐Trifft nicht zu ☐Trifft überhaupt nicht zu ☐Weiß nicht

Ich traue mir zu eine Nasenendoskopie durchzuführen und die wichtigsten anatomischen Landmarken zu benennen.

☐Trifft sehr zu ☐Trifft zu ☐Trifft nicht zu ☐Trifft überhaupt nicht zu ☐Weiß nicht

Ich traue mir zu eine Septumdeviation zu erkennen.

☐Trifft sehr zu ☐Trifft zu ☐Trifft nicht zu ☐Trifft überhaupt nicht zu ☐Weiß nicht

Ich traue mir zu ein Septumhämatom zu erkennen.

☐Trifft sehr zu ☐Trifft zu ☐Trifft nicht zu ☐Trifft überhaupt nicht zu ☐Weiß nicht

Ich traue mir zu eine polyposis nasi zu erkennen.

☐Trifft sehr zu ☐Trifft zu ☐Trifft nicht zu ☐Trifft überhaupt nicht zu ☐Weiß nicht

Ich traue mir zu eine Mundhöhle zu untersuchen und die wichtigsten anatomischen Landmarken zu benennen.

☐Trifft sehr zu ☐Trifft zu ☐Trifft nicht zu ☐Trifft überhaupt nicht zu ☐Weiß nicht

Ich traue mir zu die wichtigsten Hirnnerven, welche die Mundhöhle und den Pharynx betreffen zu benennen und zu überprüfen.

☐Trifft sehr zu ☐Trifft zu ☐Trifft nicht zu ☐Trifft überhaupt nicht zu ☐Weiß nicht

Ich traue mir zu eine akute Parotits zu erkennen.

☐Trifft sehr zu ☐Trifft zu ☐Trifft nicht zu ☐Trifft überhaupt nicht zu ☐Weiß nicht

Ich traue mir zu eine akute Tonsillits zu erkennen.

☐Trifft sehr zu ☐Trifft zu ☐Trifft nicht zu ☐Trifft überhaupt nicht zu ☐Weiß nicht

Ich traue mir zu eine flexible endonasale Endoskopie durchzuführen und die wichtigsten anatomischen Landmarken zu benennen.

☐Trifft sehr zu ☐Trifft zu ☐Trifft nicht zu ☐Trifft überhaupt nicht zu ☐Weiß nicht

Ich traue mir zu eine Stimmlippenparese zu erkennen.

☐Trifft sehr zu ☐Trifft zu ☐Trifft nicht zu ☐Trifft überhaupt nicht zu ☐Weiß nicht

Ich traue mir zu einen Hals zu untersuchen und die wichtigsten anatomischen Landmarken zu benennen.

☐Trifft sehr zu ☐Trifft zu ☐Trifft nicht zu ☐Trifft überhaupt nicht zu ☐Weiß nicht

Ich traue mir zu die wichtigsten Hirnnerven, welche das Gesicht und den Hals betreffen zu benennen und klinisch zu testen.

☐Trifft sehr zu ☐Trifft zu ☐Trifft nicht zu ☐Trifft überhaupt nicht zu ☐Weiß nicht

Ich traue mir zu eine cervicale Lymphadenopathie zu erkennen.

☐Trifft sehr zu ☐Trifft zu ☐Trifft nicht zu ☐Trifft überhaupt nicht zu ☐Weiß nicht

Ich traue mir zu im Notfall eine Koniotomie durchzuführen.

☐Trifft sehr zu ☐Trifft zu ☐Trifft nicht zu ☐Trifft überhaupt nicht zu ☐Weiß nicht
